# Supplementary material for: Patterns of Intron Gain and Loss in Fungi
Source: PLoS Biol. 2004 Nov 30;2(12):e422. doi: 10.1371/journal.pbio.0020422 (PMC532390; doi:10.1371/journal.pbio.0020422)
Supplement: Table S1 — Also available at http://genes.mit.edu/NielsenEtAl/. (4.3 MB ZIP). [file pbio.0020422.st001.zip › NielsenEtAl/html/134.html]

AN5604.1.NCU04797.1.MG08895.1.FG09280.1


```
 CLUSTAL W (1.82) Multiple Sequence Alignments - Introns Inserted


Sequence 1: NCU04797.1	350 aa
Sequence 2: FG09280.1	342 aa
Sequence 3: MG08895.1	344 aa
Sequence 4: AN5604.1	355 aa
Alignment Length: 358 aa
Number Identitical Residues: 237 aa
Alignment Score (without introns) 9962


MG08895.1 	MAD-SNN--GPET--EKVNTNIVTLTRFLTEEQAKHKEATGDFT2LLCHALQFSFKAIAY
NCU04797.1	MSTFTTNGAGAENDGEKVNTHIVTLTRFLTEEQVKHKEATGDFT2LLCHALQFSFKSIAY
FG09280.1 	MS--TTN-SGQES--EKVNTNIVTLTRFLTEEQAKHPEATGDFT2LLCHALQFSFKSIAY
AN5604.1  	MTTNSTGNGGAVGQ-ENINTDIVTLSRFFTEEQTKVPEATGDFT2LLCHALQFSFKSIAY
          	*:  :....*   . *::**.****:**:****.*  ******* ***********:***

MG08895.1 	YIRRATLVNLTGLAGSSNTTGDDQKKLDVIGNDLFIEAMRSSGKCALLVSEEEEEIIFFT
NCU04797.1	YIRRATLVNLTGLAGSSNTTGDEQKKLDVISNDLFIEAMRSSGKCALLVSEEEEHVIYFK
FG09280.1 	YIRRATLVNLTGLAGSSNITGDDQKKLDVISNDLFIEAMRSSGKCALLVSEEEDEIIYFK
AN5604.1  	YIRRASLINLTGLAGSSNTTGDDQKKLDVIGNDLFVSAMRTSGKCRILVSEEEEEAIIFD
          	*****:*:********** ***:*******.****:.***:**** :******:. * * 

MG08895.1 	NGNGSARYAVACDPIDGSSNLDAGVSVGTIFGIHKLAEGSTG-----SKEDILKPGSELL
NCU04797.1	DAK-NARYAVACDPIDGSSNLDAGVSVGTIFAIHKLAEDSTG-----TKEDILKPGTELV
FG09280.1 	DAS-DARYAVACDPIDGSSNLDAGVSVGTIFAIHKLPEGSKG-----CKEDILKPGTELL
AN5604.1  	EHP-NARYAVVCDPIDGSSNLDAGVSVGTIFGIFRLPDDILGPGKKVTAKDVLLPGTEMV
          	:   .*****.********************.*.:*.:.  *....   :*:* **:*::

MG08895.1 	AAGFTMYGASAQLVLTMKNGGVNGFTLDNAFGEFILTHPDMRMPKSRAIYSVNEGNSLYW
NCU04797.1	AAGFTMYGASAQLVITMKGGSVNGFTLDQGVGEFILTHPNMTIPRKRSIYSVNEGNSLYW
FG09280.1 	AAGFTMYGASAQLVITMRGGTVNGFTLDNGVGEFILSHPNMRLPKSRDIYSCNEGNSLYW
AN5604.1  	AAGFTMYGASAQLVITMKNGGVNGFTLENSLGEFILTHPNMKLPPSRAIYSVNEGNSMYW
          	**************:**:.* ******::..*****:**:* :* .* *** *****:**

MG08895.1 	EDHVKEYFSSLKHPQEKSGKPYSARYIGSMVADAYRTLLYGGIFAYPADKKSPKGKLRIL
NCU04797.1	EDDVKAYFNSLKETQEDSGKPYSARYIGSMVADAYRTLLYGGMFAYPADKKSPKGKLRIL
FG09280.1 	DDKTINYFNSLKQAQEN-GKPYSARYIGSMVADAYRTLLYGGIFAYPADKKSPKGKLRIL
AN5604.1  	DDWCNAYFDSLKRPGEG-KKPYSARYIGSMVADAYRTLLYGGVFAYPADSKSPKGKLRIL
          	:*    **.***.. *   ***********************:******.**********

MG08895.1 	YECAPMAMVFENA1GGQAIDSNMKRMLDVVPEHIHDRSGIFMGSWDEVEKVKAYHK----
NCU04797.1	YECAPMAMIFENA1GGQAVDSKMNRMLEVVPEHIHDKAGIFMGSYDEVEKVKKYHGKA--
FG09280.1 	YECAPMALIFENA1GGQAVDSKMNRLLEVVPEHIHDKAGIFMGSYDEVEKVKKFHQ----
AN5604.1  	YECAPMAMVFENA~GGKAVNSRMERLLSVQPESIHDKSGVFLGSRDEVQKVIDTYNKYKK
          	*******::**** **:*::*.*:*:*.* ** ***::*:*:** ***:**   : . ..

NCU04797.1	
FG09280.1 	
MG08895.1 	
AN5604.1  	
          	
```
